# Postnatal Gene Therapy Improves Spatial Learning Despite the Presence of Neuronal Ectopia in a Model of Neuronal Migration Disorder

**DOI:** 10.3390/genes7120105

**Published:** 2016-11-29

**Authors:** Huaiyu Hu, Yu Liu, Kevin Bampoe, Yonglin He, Miao Yu

**Affiliations:** Department of Neuroscience and Physiology, Center for Vision Research, Department of Ophthalmology, State University of New York Upstate Medical University, Syracuse, NY 13210, USA; liuyu@upstate.edu (Y.L.); bampoek@upstate.edu (K.B.); heyonglinhyl@163.com (Y.H.); yum@upstate.edu (M.Y.)

**Keywords:** neuronal migration disorder, type II lissencephaly, adeno-associated virus, gene therapy

## Abstract

Patients with type II lissencephaly, a neuronal migration disorder with ectopic neurons, suffer from severe mental retardation, including learning deficits. There is no effective therapy to prevent or correct the formation of neuronal ectopia, which is presumed to cause cognitive deficits. We hypothesized that learning deficits were not solely caused by neuronal ectopia and that postnatal gene therapy could improve learning without correcting the neuronal ectopia formed during fetal development. To test this hypothesis, we evaluated spatial learning of cerebral cortex-specific protein *O*-mannosyltransferase 2 (POMT2, an enzyme required for *O*-mannosyl glycosylation) knockout mice and compared to the knockout mice that were injected with an adeno-associated viral vector (AAV) encoding POMT2 into the postnatal brains with Barnes maze. The data showed that the knockout mice exhibited reduced glycosylation in the cerebral cortex, reduced dendritic spine density on CA1 neurons, and increased latency to the target hole in the Barnes maze, indicating learning deficits. Postnatal gene therapy restored functional glycosylation, rescued dendritic spine defects, and improved performance on the Barnes maze by the knockout mice even though neuronal ectopia was not corrected. These results indicate that postnatal gene therapy improves spatial learning despite the presence of neuronal ectopia.

## 1. Introduction

Neuronal migration disorders during development result in brain malformations and cause the patients to suffer from epilepsy and severe mental retardation, including learning deficits. Type II lissencephaly, characterized by overmigration of neurons during development of the cerebral cortex, is found in a group of congenital muscular dystrophies such as Walker–Warburg syndrome and muscle-eye-brain disease. These genetic diseases are often caused by mutations in genes encoding glycosyltransferases (or putative glycosyltransferases) such as *POMT1* and *POMT2* (encoding protein *O*-mannosyltransferases 1 and 2, respectively) [1,2,3]; *POMGnT1* (encoding protein *O*-mannose *N*-acetylglucosaminyl transferase 1) [4]; *LARGE* (encoding like-glycosyltransferase) [5]; and others including fukutin and fukutin-related protein (FKRP) [6,7,8,9,10,11,12,13,14]. *POMT1* and *POMT2* are required for the initiation of *O*-mannosyl glycosylation [15,16]. LARGE synthesizes a sugar branch on *O*-linked mannose consisting of repeating disaccharide units of [–3-xylose–α1,3-glucuronic acid-β1–] [17] that bind extracellular matrix molecules such as laminin. A common molecular consequence of these mutations is the hypoglycosylation of α-dystroglycan (α-DG), which affects its binding to extracellular matrix molecules [18]. There is no effective therapy for these diseases.

Previous research using mouse models of *O*-mannosyl glycosylation defects has shown promising results in treating muscular dystrophy by gene therapy. Overexpression of LARGE via an adenoviral vector is capable of hyperglycosylating skeletal muscle α-DG in vivo [19]. Although LARGE overexpression by transgenic approach exacerbates muscular dystrophy in FKRP knock-down mice [20] and fukutin knockout mice [21] thought to be caused by inhibition of regeneration from satellite cells, we have shown that LARGE expression ameliorates muscular dystrophy in LARGE mutant and POMGnT1 knockout mice when delivered systemically by an adeno-associated viral vector 9 (AAV9) after birth [22]. Furthermore, AAV-mediated expression of LARGE, fukutin-related protein, or B4GALNT2 (GALGT2) ameliorates muscular dystrophic phenotype in FKRP mutant mice [23,24,25] and in a mouse model bearing a pathogenic human FKRP mutation [26]. The benefit of *LARGE* gene therapy requires endogenous fukutin [27]. Although postnatal gene therapy ameliorates muscular dystrophic phenotype in several mouse mutant models, whether it can improve brain dysfunction is unknown.

During brain development in mouse models of *O*-mannosyl glycosylation defects, hypoglycosylation of α-DG results in disruptions of the pial basement membrane, which causes neuronal migration defects that include overmigration of neurons in the neocortex, granule cell ectopia in the dentate gyrus, and failure of migration of some cerebellar granule cells [28,29,30,31]. It is believed that neuronal ectopia is the primary cause of brain dysfunction and that prenatal intervention is necessary to treat abnormal neuronal migration and improve brain function [32]. However, prenatal gene therapy is technically challenging and ethically controversial. In this report, we tested the hypotheses that spatial learning deficit was at least partially brought about by altered dendritic spine plasticity and that repair of spine plasticity enhances spatial learning in spite of neuronal ectopia. We chose to use cerebral cortex-specific POMT2 knockout mice to test the hypotheses because these mice lacked muscular defects, and thus behavioral assays would not be influenced by muscular defects. Our results indicated that cerebral cortex-specific POMT2 knockout caused dendritic spine defects and that postnatal gene therapy in the brain improved spatial learning despite the presence of neuronal ectopia.

## 2. Materials and Methods

### 2.1. Animals

Emx1-Cre transgenic mice [33] were obtained from the Jackson Laboratories (Bar Harbor, ME, USA). POMT2-floxed mice have been reported [28]. These mice were purposely maintained on a 129SV and C57/B6 hybrid background to avoid artificial phenotypes that can be caused by homozygosity of inbred strains. Protocols for using laboratory mice were approved by the Institutional Committee for Use and Care of Laboratory Animals at State University of New York Upstate Medical University (CHUA#367) and adhered to the guidelines of the National Institutes of Health. All efforts were made to minimize pain and suffering of animals.

### 2.2. Viral Vectors and Injection

Adeno-associated viral vectors (serotype 9) for expression of enhanced green fluorescent protein (EGFP) (AAV9-EGFP, 3.2 × 10^13^ GC/mL) and c-Myc-tagged POMT2 (AAV9-POMT2, 3.4 × 10^13^ GC/mL), driven by the chicken β-actin promoter, were constructed at Vector Biolabs (Philadelphia, PA, USA) on a fee-for-service basis. AAV9-POMT2 or AAV9-POMT2 and AAV9-EGFP mixtures (10:1, 3 μL) were injected into the lateral ventricles of animals three to four days postnatally using pooled capillary tubes. Control mice were injected with equal volumes of phosphate-buffered saline (PBS) or PBS-diluted AAV9-EGFP (1:10).

### 2.3. Antibodies and Wisteria Floribunda Lectin

Antibodies were obtained as follows: Monoclonal anti-β-DG (43DAG1/8D5) from Leica Biosystems (Cat#B-DG-CE, Buffalo Grove, IL, USA); polyclonal anti-Tbr1 antibody from Abcam (Cat#ab31940, Cambridge, MA, USA); polyclonal anti-laminin-1 from Sigma-Aldrich (Cat#L9393, St. Louis, MO, USA); Monoclonal IIH6C4 from Millipore Corporation (Cat#05-593, Billerica, MA, USA); monoclonal anti-c-Myc from ThermoFisher Scientific (Cat#MA1-980, Rockford, IL, USA); and polyclonal anti-POMT2 from Atlas Antibodies (Cat#HPA003663, Bromma, Sweden). FITC-conjugated wisteria floribunda lectin (WFA) was obtained from EY Laboratories (Cat#F3101-2, San Mateo, CA, USA).

### 2.4. Western Blot Analysis and Ligand Overlay Experiments

Neocortex was homogenized in lysis buffer (50 mM Tris-HCl, pH 7.4, 150 mM NaCl, 1% Triton X-100) with a protease inhibitor cocktail (Roche Diagnostics, Indianapolis, IN, USA). After centrifugation at 16,100× *g* for 20 min at 4 °C, the supernatants were collected. To isolate glycoproteins, 50 μL wheat germ agglutinin (WGA)-affinity gel (Cat#A-2101-5, EY Laboratories) was mixed with 2 mg of lysate proteins and incubated for 4 h at 4 °C. The WGA-gel was then washed three times with the lysis buffer. Bound glycoproteins were eluted by sodium dodecyl sulfate polyacrylamide gel electrophoresis (SDS-PAGE) loading dye, separated on SDS-PAGE, and electrotransferred onto polyvinylidene fluoride (PVDF) membranes. For immunodetection with antibodies, the PVDF membranes were blocked with 3% bovine serum albumin (BSA) in TBST (50 mM Tris, pH 7.4, 150 mM NaCl, 0.05% Tween-20) for 30 min and incubated with primary antibodies in TBST containing 3% BSA for 2 h and washed with TBST. The membranes were then incubated with goat anti-mouse IgG (or IgM when appropriate, Jackson ImmunoResearch Laboratories, West Grove, PA, USA) conjugated with horseradish peroxidase (1:3000) for 45 min. After extensive washing with TBST, the signal was visualized with SuperSignal west pico chemiluminescent substrate (ThermoFisher Scientific).

For laminin overlay assay, the PVDF membrane was incubated with 3% BSA in Tris-buffered saline (50 mM Tris, pH7.4, 150 mM NaCl, 1 mM CaCl_2_, and 1 mM MgCl_2_) laminin-111 overnight at 4 °C. Immunodetection of bound laminin is identical to the above procedure with the exception that all buffers contained 1 mM CaCl_2_ and 1 mm MgCl_2_. After washing with TBST, the membrane was incubated with a rabbit antibody against laminin-1 (Sigma-Aldrich, 1:2000) for 2 h and washed with TBST. The membrane was then incubated with goat anti-rabbit IgG conjugated with horseradish peroxidase (Jackson ImmunoResearch Laboratories, 1:3000) for 45 min. After extensive washing with TBST, the signal was visualized with SuperSignal west pico chemiluminescent substrate. WGA preferentially binds to glycans bearing *N*-acetylglucosamine and sialic acid. Isolation of WGA-binding glycoproteins enriches for IIH6C4 immunoreactive and laminin binding-α-DG for evaluation of functional glycosylation but may miss glycans that are not recognized by WGA on other proteins.

### 2.5. Fluorescence Staining

Brains were fixed by 4% paraformaldehyde, cryo-protected with 30% sucrose, and cryo-sectioned (10 µm thickness) in the coronal plane. The sections were permeabilized with 0.1% Triton X-100 in phosphate buffer (PB). Slides were blocked with 3% BSA in 0.1 M PB at room temperature for 1 h in a humidified environment. Anti-Tbr1 antibody was used at 1:500 dilution. After washing with PB containing 0.1% Triton X-100, the slides were incubated with FITC-conjugated goat anti-rabbit IgG (Jackson ImmunoResearch 1:300) for 2 h at room temperature in a humidified environment. Counterstaining with DAPI (Sigma-Aldrich) was used to visualize the nuclei. After washing with PB three times, the sections were covered with VECTASHIELD^®^ mounting medium (Vector Laboratories, Burlingame, CA, USA. Cat# H-1000) by coverslips. Direct FITC-conjugated WFA staining was carried out similarly without incubating with a secondary antibody. To visualize fluorescence, a Zeiss Axioskop epifluorescence microscope (Cambridge, UK) was used. Epifluorescence images were captured with QCapture Pro 6.0 (QImaging, Surrey, BC, Canada).

To evaluate the distribution of T-box brain protein 1(Tbr1)-positive nuclei and WFA-labeled neurons in the neocortex, every 10th section from the forebrain was collected for staining. For Tbr1-positive neurons, images were taken at the pial surface directly dorsal to the lateral ventricle on the sections at the level of anterior commissure with a 40× objective. Tbr1-positive nuclei were counted on the images. For WFA-labeled neurons, images were obtained with a 5× objective to cover the entire neocortical wall at the level of anterior commissure. A region spanning 500 µm of the cortex (medial-lateral) directly dorsal to the lateral ventricles from the pial surface to the white matter was divided into 5 equal compartments. WFA-labeled neurons in each compartment were counted.

### 2.6. Behavioral Test

For Barnes maze test, all mice were analyzed on a Barnes maze (Stoelting, Wood Dale, IL, USA) between three and five months of age. Animals of different treatment groups were matched by sex and age. The day before training trials, each mouse was habituated to the maze environment by being placed at the center of the maze and shielded with a cylindrical black chamber. After 10 s, the chamber was lifted and the mouse was gently guided to enter the target escape hole and was allowed to stay in the hole for 2 min. During the training period, each animal received eight sessions of training (one session/day) with four trials per session at a 15 min interval between trials. On each trial the mouse was placed at the center of the maze as described above. After 10 s, the chamber was lifted and a buzzer (electronic metronome, IMT-400, Intelli, New Market, VA, USA) switched on to produce a noise of 85 dB as a motivator. The trial ended when the mouse entered the target hole. If the mouse did not enter within 3 min, it was guided to the target hole gently. The buzzer was turned off immediately after the mouse entered the hole. The mouse was allowed to stay in the hole for 1 min before being returned to its home cage. To avoid interference by olfactory cues, the maze was cleaned with 10% alcohol to dissipate odor cues and to provide a standard olfactory context for each trial. Latency to the target hole was obtained from the video recordings and averaged in blocks of trials each day. To assess memory retention, probe trials were conducted on day 9 to determine if the animal remembered the location of the target hole. The mouse was placed at the center of the maze shielded with the chamber and the target hole was closed. After 10 s, the chamber was lifted and the buzzer switched on. The mouse was allowed to explore the maze for 90 s. The number of pokes in each hole was recorded.

For elevated plus maze, the mice were placed at the center of the maze and left in the maze for 5 min. The times spent in open and closed arms were recorded.

### 2.7. Analysis of Dendrites and Spines

The brains of five- to six-month old cerebral cortex-specific POMT2 knockout mice were fixed and cut in accordance with stereological principles [34,35] into 200 μm sections in the coronal plane. Consecutive sections throughout the entire hippocampus were collected and every third section was mounted onto one set of slides. All three sets of slides were subjected to Golgi-Cox staining in parallel (Bioenno, Irvine, CA, USA). One set of sections was used to carry out unbiased systematic sampling throughout the entire anterior-posterior extent of the hippocampus. Eight intact CA1 neurons were selected per animal. Selected cells were captured, reconstructed, and drawn using a Nikon E400 microscope with Neurolucida Version 10 (MBF Bioscience, Williston, VT, USA). Branching of basal and apical dendrites was assessed using Sholl analysis [36], which measures the total dendritic length and number of intersections at concentric circles at 10 μm intervals from the soma center. For spine analysis, the basal (in stratum oriens), apical stem and apical oblique branches (in stratum radiatum) and distal branches (in stratum lacunosum moleculare) of eight randomly selected neurons were imaged with a 100× objective. Two to four basal dendrites per neuron were imaged. For apical dendrites, the stem, the third to fifth oblique, and two to four distal branches per neuron were imaged. Spines were characterized into mushroom, thin, and stubby types [37,38]. Filopodia spines were rarely observed and thus they were not included in the analysis. Sholl and spine analyses were carried out blind.

### 2.8. Statistical Analysis

Latencies to the target hole were analyzed by repeated measures analysis of variance (ANOVA) followed by post-hoc pair wise comparison between groups with Bonferroni correction. Elevated plus maze data were analyzed by Student’s *t* test. Data from cell counting and spine analysis were evaluated by ANOVA or Student’s *t* test.

## 3. Results

**Figure 1:**
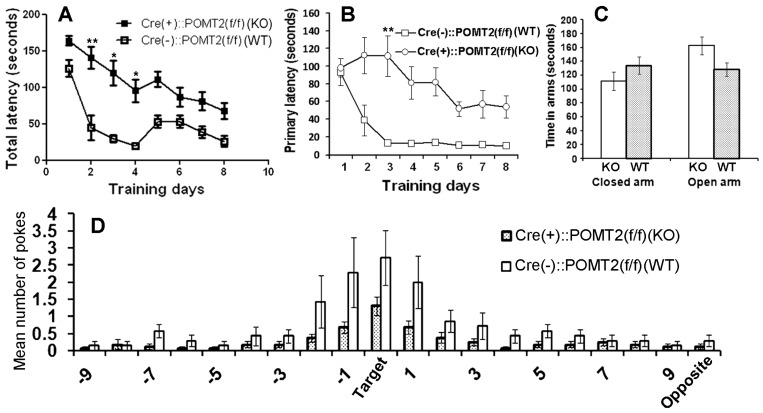
Cerebral cortex-specific protein *O*-mannosyltransferase 2 (POMT2) knockout mice [Emx1-Cre(+)::POMT2(f/f)] exhibit spatial learning deficits. Cerebral cortex specific POMT2 knockout mice (*n* = 10) and wildtype (Cre-negative) littermate control mice (*n* = 8, 3–5 months old) were tested for their performance on the Barnes maze task. (**A**) The knockout animals took a longer time to enter the target hole than the littermate controls (*p* = 0.0043, repeated measures analysis of variance (ANOVA)). ** *p* < 0.01; * *p* < 0.05, post-hoc Bonferroni comparisons test. (**B**) Primary latency was increased in the knockout animals (*p* = 0.0114, repeated measures ANOVA). ** *p* < 0.01, post-hoc Bonferroni comparisons test. (**C**) Cerebral cortex-specific POMT2 knockout mice did not exhibit increased anxiety on the elevated plus maze. (**D**) In the probe trial, the knockout mice visited the target hole and the holes immediately adjacent to the target hole (+1 and −1) fewer times than the wildtype mice did. Abbreviations: KO, knockout; WT, wildtype.

In cerebral cortex-specific POMT2 knockout mice [Emx1-Cre(+)::POMT2(f/f)] where POMT2 is deleted in projection neurons and glial cells of the cerebral cortex [28,33], overmigration of neurons occurs during cortical development. This results in several structural abnormalities because of neuronal ectopia, including neuronal lamination defects in the neocortex, fused hemispheres, and a dentate gyrus with abnormal morphology [28]. To determine whether these mice exhibit cognitive deficits, we performed a Barnes maze test [39]. The knockout mice exhibited an increase in total latency to the target hole (time for mice to enter the target hole) compared to the wildtype (Cre-negative) animals (Figure 1A, *p* = 0.0043, repeated measures ANOVA). Primary latency to the target hole (time for mice to poke on the target hole for the first time during a trial) was also increased in the knockout animals (Figure 1B, *p* = 0.0114, repeated measures ANOVA). During the probe trial (Figure 1D), the knockout animals poked the target hole and the two holes immediately left (−1) or right (+1) of the target hole fewer times than the wildtype mice. The increased latency to the target hole in the knockout mice could be caused by an increase in anxiety. To determine if anxiety contributed to the increased latency to the target hole in the Barnes maze, we evaluated the knockout mice on an elevated plus maze. Mice with anxiety were expected to spend more time in the closed arms and less time in open arms than wildtype control mice. However, the time spent in closed and open arms by the knockout mice was not significantly different from the wildtype controls (Figure 1C, *p* = 0.22 and 0.054 for closed and open arms respectively, two-tailed Student’s *t* test), indicating that the knockout mice did not exhibit increased anxiety. These results indicate that cerebral cortex-specific POMT2 knockout mice exhibited learning deficits.

**Figure 2:**
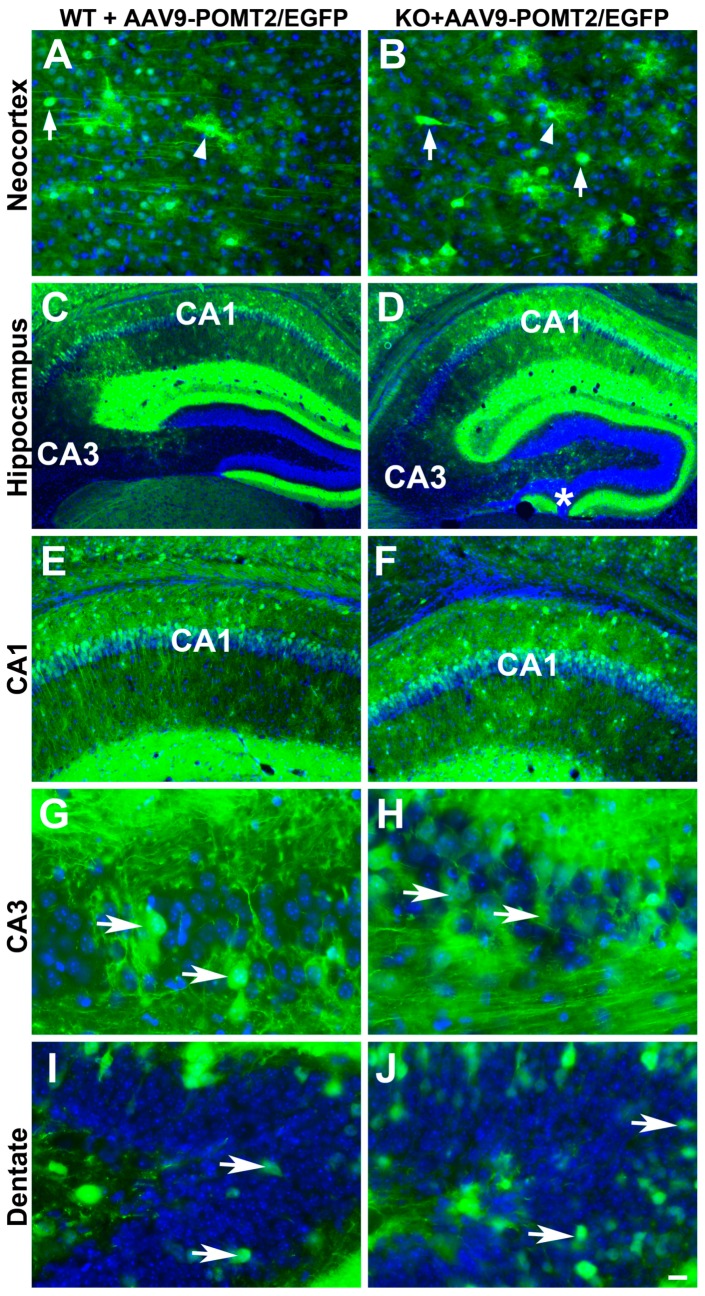
Neocortical and hippocampal neurons were efficiently transduced by the viral vector in wildtype and cerebral cortex-specific POMT2 knockout animals. GFP fluorescence micrographs of cerebral cortex-specific knockout and wildtype mice injected with viral mixtures of adeno-associated viral vector 9 (AAV9)-POMT2 and AAV9-EGFP at birth and analyzed at five months of age. (**A**,**B**) Examples of labeled neurons (arrows) and astrocytes (arrowheads) in the neocortex of wildtype and knockout mice. (**C**,**D**) EGFP fluorescence in the hippocampus; (**E**,**F**) CA1 region of the hippocampus. (**G**,**H**) CA3 region of the hippocampus; (**I**,**J**) Dentate gyrus. Abbreviations: KO, knockout; WT, wildtype. Scale bar: 25 μm for (**A**,**B**,**G**,**H**); 100 μm for (**E**,**F**); 200 μm for (**G**,**H**).

To determine whether deletion of POMT2 affects gene delivery by AAV9 vectors, we constructed adeno-associated viral vectors for the expression of EGFP and POMT2 driven by the chicken β-actin promoter, AAV9-EGFP and AAV9-POMT2 vectors. We injected a mixture of AAV9-POMT2 and AAV9-EGFP at a 10:1 ratio into the lateral ventricles of newborn wildtype and cerebral cortex-specific POMT2 knockout mice. Co-injection of AAV9-EGFP would allow us to evaluate the types of neurons transduced and whether *O*-mannosyl glycosylation deficiency affects AAV9 gene delivery. Control injections were AAV9-EGFP suspended in PBS or PBS alone. We then examined AAV transduction in the neocortex and hippocampus of these animals at adulthood (Figure 2). EGFP labeled neurons (arrows) and astrocytes (arrowheads) were frequently found in the neocortex of both wildtype and knockout animals (Figure 2A,B). Extensive labeling of neurons in the hippocampus (Figure 2C,D) was observed including pyramidal neurons of CA1 (Figure 2E,F) and CA3 (Figure 2G,H), and granule cells of the dentate gyrus (Figure 2I,J) of wildtype and knockout animals. Similar distribution patterns of EGFP-labeled neurons were observed in three wildtype and four knockout animals. Thus, efficient labeling of neurons was observed in the cerebral cortex and hippocampus of the wildtype and knockout mice, indicating that lack of *O*-mannosyl glycans did not affect gene transduction efficiency in the knockout brain.

**Figure 3:**
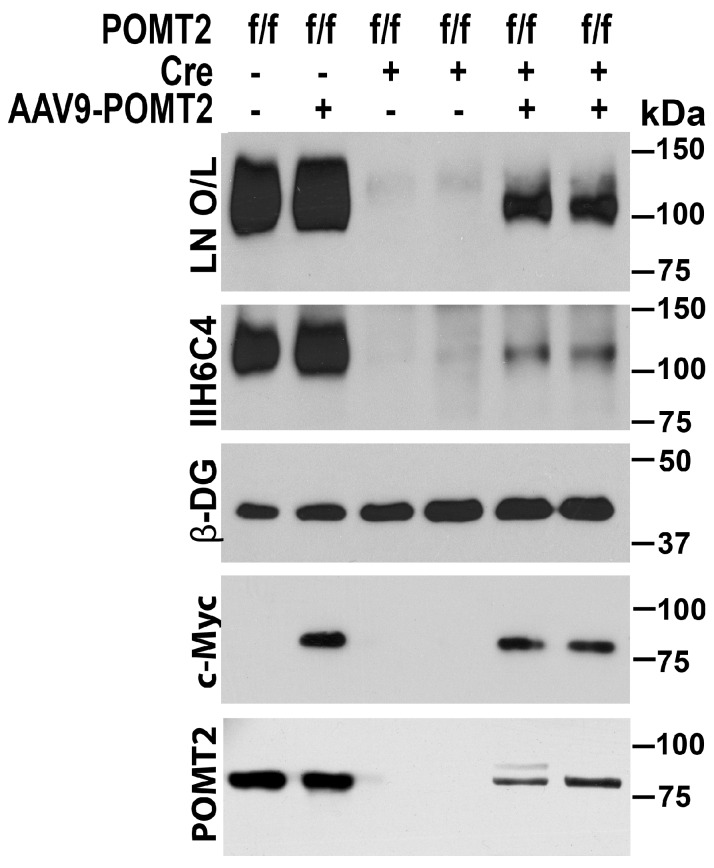
Rescue of *O*-mannosyl glycosylation defects by AAV9-POMT2-treatment. Western blot with anti-β-DG and IIH6C4 antibodies and laminin overlay (LN O/L) of neocortical glycoproteins isolated with WGA agarose. Wildtype and AAV9-POMT2 treated wildtype samples showed same levels of IIH6C4 immunoreactivity and laminin binding at 120 kDa. Both untreated knockout samples exhibited only remnant levels of IIH6C4 immunoreactivity and laminin binding. The remaining IIH6C4 immunoreactivity is likely from local circuit neurons and blood vessels. Both AAV9-POMT2 treated knockout samples showed significantly increased IIH6C4 immunoreactivity and laminin binding over the untreated knockouts. As control, β-DG was readily detected in all samples with anti-β-DG. Anti-c-Myc detected viral vector-expressed POMT2 in the lysates of AAV9-POMT2 treated samples only. Weak anti-POMT2 immunoreactivity was detected in the lysates of AAV9-POMT2 treated but not untreated knockout neocortex. These results indicate that AAV9-POMT2 treatment in the brain recovers functional glycosylation of α-DG in the POMT2 knockout mice.

To evaluate whether AAV9-POMT2 injection restored glycosylation of α-DG, we carried out an immunoblot with the IIH6C4 antibody, which recognizes functional glycosylation of α-DG, on glycoproteins isolated from the neocortex with WGA agarose (Figure 3). IIH6C4 immunoreactivity was readily detected in the wildtype neocortex with or without AAV9-POMT2 treatment. The untreated knockout neocortex exhibited only remnant levels of IIH6C4 immunoreactivity. However, AAV9-POMT2 injection significantly increased the amount of IIH6C4 immunoreactivity in the knockout neocortex.

*O*-mannosyl glycosylation is essential for α-DG binding to extracellular matrix. Hypoglycosylation of α-DG affects its binding to extracellular matrix molecules including laminin [40,41,42], agrin [43], perlecan [44], neurexin [45], Slit [46], and pikachurin [47,48,49]. To evaluate the effect of AAV9-POMT2 treatment on extracellular matrix binding activity of dystroglycan in the knockout brain, we carried out laminin overlay assays. Laminin binding by α-DG was significantly reduced in the knockout neocortex compared to wildtype controls as indicated by the laminin overlay assay. AAV9-POMT2 treatment significantly increased laminin binding in the knockout neocortex. Thus, AAV9-POMT2 treatment resulted in partial restoration of functional glycosylation of α-DG in the cerebral cortex-specific POMT2 knockout mice but did not change glycosylation in the wildtype controls.

**Figure 4:**
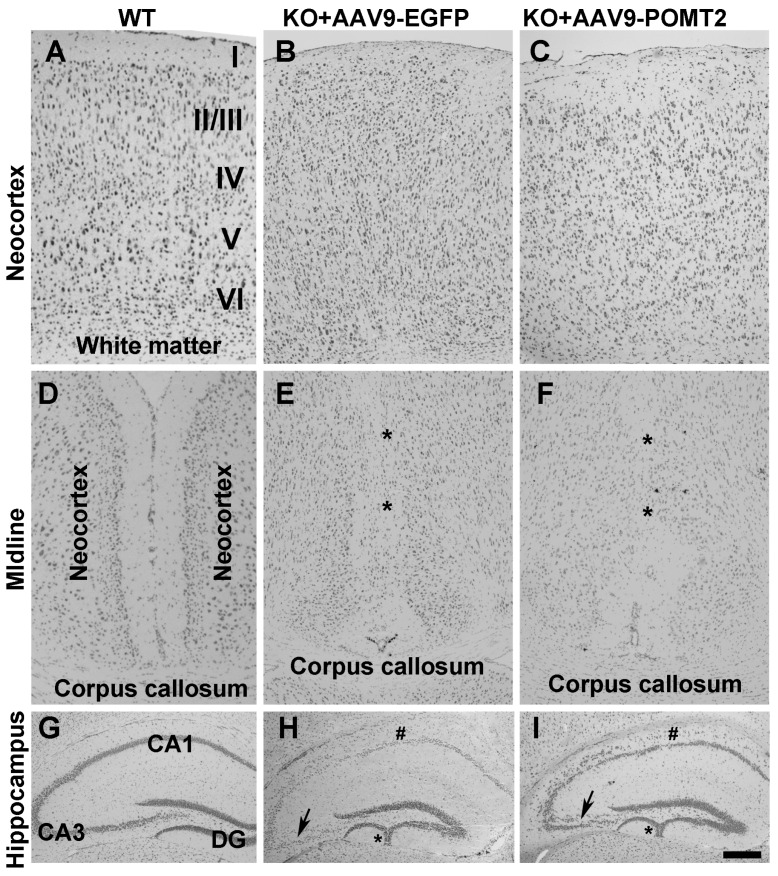
Histological abnormalities of the knockout mice were not rescued by postnatal AAV9-POMT2 treatment. Brains of AAV9-EGFP- and AAV9-POMT2-treated mice at three to five months of age were sectioned in the coronal plane and stained with cresyl violet. (**A**–**C**) Neocortex of AAV9-EGFP- and AAV9-POMT2-treated wildtype mice and knockout mice. Lamination defects were present in the knockout neocortex treated with either AAV9-EGFP or AAV9-POMT2. (**D**–**F**) Midline of two cerebral hemispheres of AAV9-EGFP- and AAV9-POMT2-treated wildtype mice and knockout mice. Two hemispheres were fused in the knockout neocortex treated with either AAV9-EGFP or AAV9-POMT2 (Asterisks in E and F). (**G**–**I**) Hippocampus of AAV9-EGFP- and AAV9-POMT2-treated wildtype mice and knockout mice. Lamination defects of neurons in CA1, CA3, and dentate gyrus was present in the knockout neocortex treated with either AAV9-EGFP or AAV9-POMT2. Asterisks in H and I indicate granule cell ectopia and # signs indicate lamination defects of CA1. Abbreviations: DG, dystroglycan; KO, knockout; WT, wildtype. Scale bar: 100 µm for (**A**–**F**); 200 µm for (**G**–**I**).

The postnatal injection of AAV9-POMT2 was not expected to rescue the abnormal migration phenotype because overmigration of neocortical neurons starts at Embryonic Day 13.5 in the knockout animals [28] while gene therapy was delivered after birth. Indeed, the characteristic neuronal ectopia phenotypes were not rescued in the knockout mice despite AAV9-POMT2 treatment (Figure 4). In the wildtype animals, the layers of the neocortex were clearly identified (Figure 4A). However, neocortical lamination in the knockout animals with or without AAV9-POMT2 treatment was abnormal in that they exhibited no discernable layers (Figure 4B,C, *n* = 5). Similarly, fusion of hemispheres at the midline was observed in the knockout animals (asterisks, Figure 4E, *n* = 5), an abnormality remained in AAV9-POMT2 treated knockout animals (asterisks, Figure 4F, *n* = 5). In the hippocampus, the granule cell ectopia in the dentate gyrus (asterisk) and lamination defects of CA1 (# sign) and CA3 (arrows) neurons were observed in the knockout animals (Figure 4H, *n* = 5). These histological defects were also observed in all AAV9-POMT2 treated knockout animals (Figure 4I, *n* = 5).

**Figure 5:**
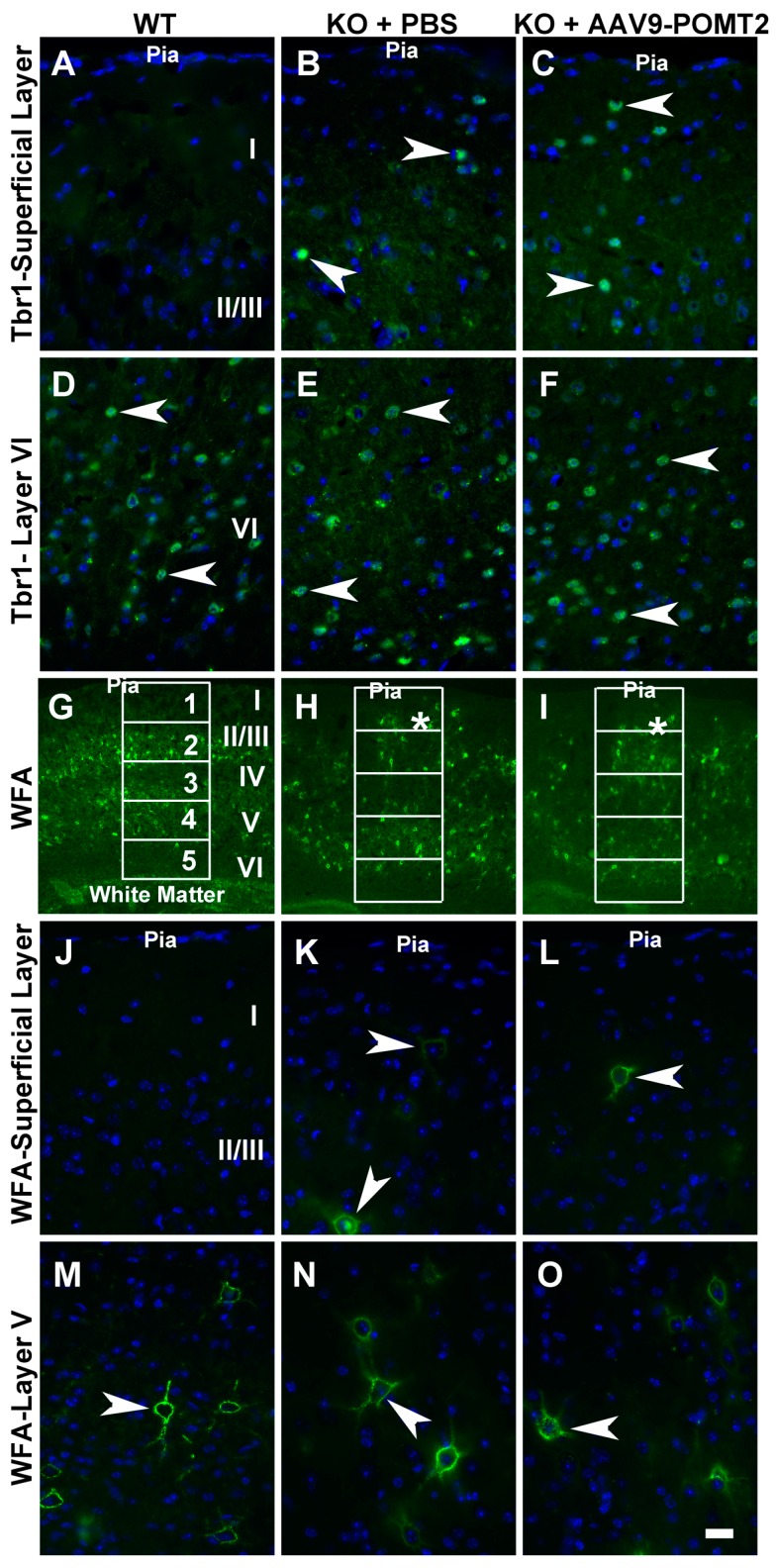
Lamination defects in the neocortex of the knockout mice were not rescued by postnatal AAV9-POMT2 treatment. Brains of PBS-treated wildtype and knockout mice and AAV9-POMT2-treated knockout mice at three to five months of age were sectioned in the coronal plane and stained with antibody against: T-box brain protein 1(Tbr1) (**A**–**F**); and wisteria floribunda lectin (WFA) (**G**–**O**). All images were from the neocortical wall at the level of the anterior commissure. (**A**–**C**) Superficial layers of wildtype, PBS-treated knockout, and AAV9-POMT2-treated knockout mice. Tbr1-positive nuclei were found at superficial regions of neocortex in the knockout mice treated with PBS or AAV9-POMT2 but were never observed in layer I–II/III of wildtype mice. (**D**–**F**) Layer VI of wildtype and corresponding locations of PBS-treated knockout and AAV9-POMT2-treated knockout mice. Tbr1-positive nuclei were observed in the wildtype mice and the knockout mice treated with PBS and AAV9-POMT2. (**G**–**I**) Neocortical wall of wildtype and PBS- or AAV9-POMT2-treated knockout mice. WFA-labeled neurons in the knockout mice were disorganized with some near the pial surface (asterisks). (**J**–**L**) Superficial layers of wildtype, PBS-treated knockout, and AAV9-POMT2-treated knockout mice. WFA-labeled neurons were rarely found near the pial of wildtype mice but frequently observed in PBS-treated and AAV9-POMT2-treated knockout mice (arrowheads). (**M**–**O**) Layer V of wildtype and corresponding location in the knockout mice. WFA-labeled neurons were clearly observed in wildtype mice and PBS- and AAV9-POMT2-treated mice. KO, knockout; WT, wildtype. Scale bar: 20 µm (**A**–**F**,**J**–**O**); 160 µm (**G**–**I**).

To further evaluate the lamination defects in the knockout neocortex with and without AAV9-POMT2 treatment, we carried out immunofluorescence staining with Tbr1 antibody, a nuclear marker for a subset of layer VI neurons and WFA, which stains mainly a subset of neurons in layers II/III to layer V (Figure 5). In wildtype neocortex, Tbr1-positive nuclei were observed in layer VI and no positive nucleus was observed in layer I (Figure 5A and arrowheads in Figure 5D). In the knockout cortex and AAV9-POMT2-treated cortex, however, Tbr1-labeled nuclei were not only observed in the deepest cortical layer (arrowheads, Figure 5E,F) but also observed in other locations including the most superficial neocortical parenchyma (arrowheads, Figure 5B,C). Unlike the wildtype, it was not possible to identify layers I, II/III, IV, V, and VI in the knockout neocortex. We, therefore, counted Tbr1-positive nuclei in layers I–II/III of wildtype cortex and corresponding regions of the knockout cortex. While no Tbr1-positive nuclei was observed in layers I–II/III of the wildtype cortex, 6.6 ± 0.26 and 6.7 ± 0.57 Tbr1-positive nuclei per 2740 µm^2^ were observed at the corresponding regions of knockout and AAV9-POMT2-treated knockout cortex, respectively. With regards to WFA staining, labeled neurons were mainly observed in layer II/III to layer V in the wildtype cortex (Figure 5G). Higher magnification showed that WFA-labeled neurons were rarely observed in layer I (Figure 5J) but were frequently observed in layer V (arrowhead, Figure 5M). In the knockout cortex, WFA-labeled neurons were disorganized with presence of labeled neurons in superficial regions (asterisk in Figure 5H). High magnification images showed that WFA-labeled neurons were frequently observed in superficial layers as well as in the location where wildtype layer V would be located (arrowheads, Figure 5K,N). In AAV9-POMT2-treated knockout neocortex, WFA-labeled neurons were distributed similarly to the PBS-treated knockout neocortex. WFA-labeled neurons were not only found in layer V but also found in superficial layers (asterisk Figure 5I,L,O). To quantify this effect, we divided the cortex into five equal compartments from the pial surface to the white matter and counted the WFA-labeled neurons in each compartment (Figure 5G–I, Table 1). The number of WFA-labeled neurons in compartment 1 (superficial compartment) was significantly increased in the knockout as well as AAV9-POMT2-treated knockout animals over the wildtype. However, the difference between the knockout mice treated with PBS and AAV9-POMT2 was not statistically significant. In addition, the disruption of the pial basement membrane and the meninges in the neocortex of the knockout mice was not rescued when evaluated by immunofluorescence staining with basement membrane marker laminin-1 and fibroblast marker ER-TR7 (data not shown). Together, these results indicate that neuronal ectopia is not rescued in the knockout mice with postnatal AAV9-POMT2 treatment.

**Figure 6:**
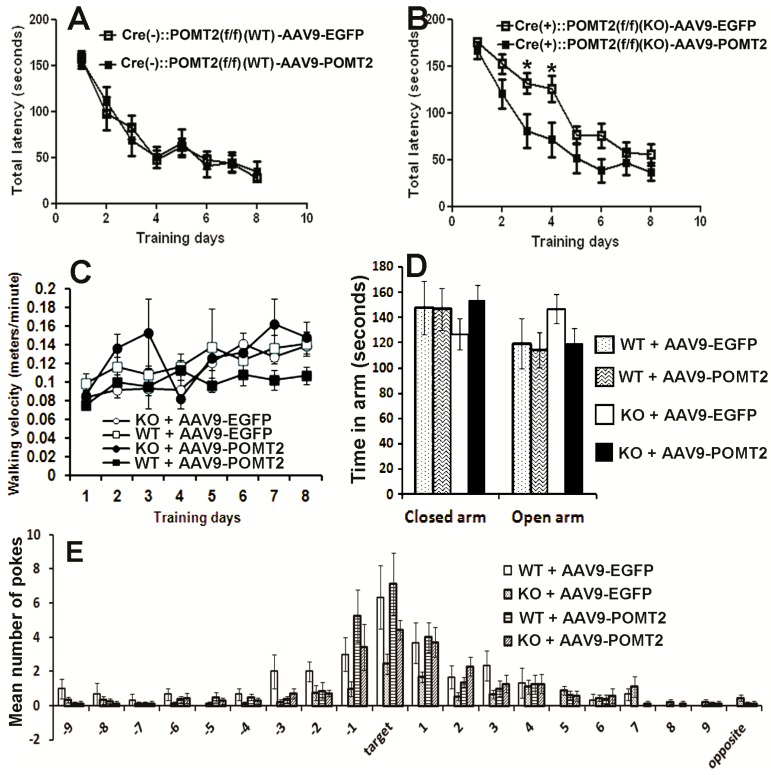
Learning deficits in cerebral cortex-specific POMT2 knockout mice are rescued by AAV9-POMT2-treatment. AAV9-EGFP- and AAV9-POMT2-treated mice were analyzed using the Barnes maze and the elevated plus maze at three to five months of age. (**A**) Wildtype animals exhibited similar latency to the target hole with (*n* = 13) or without (*n* = 11) AAV9-POMT2 treatment, indicating that AAV9-POMT2 treatment did not change spatial learning in wildtype animals. (**B**) Compared to AAV9-EGFP-treated knockout mice (*n* = 16), AAV9-POMT2-treated knockout mice (*n* = 10) showed decreased latency to the target hole (*p* = 0.0226, repeated measures ANOVA). Asterisks: *p* < 0.05, post-hoc Bonferroni comparisons test. (**C**) Walking velocity on the Barnes maze platform was not significantly different amongst different groups. (**D**) Time spent in the open and closed arms of the elevated plus maze was not significantly different in AAV9-POMT2-treated animals, indicating that there was not a change in anxiety levels after gene therapy. (**E**) The probe trial showed that AAV9-EGFP-treated knockout mice visited the target hole and the two holes adjacent to the target hole fewer times than AAV9-EGFP-treated wildtype mice. AAV9-POMT2-treatment increased the knockout mice’s number of visit to these holes compared to the AAV9-EGFP-treatment. Abbreviations: KO, wildtype; WT, wildtype.

Next, performance of AAV9-POMT2 treated animals was analyzed on the Barnes maze. In this cohort of animals, the knockout animals also exhibited prolonged latency to enter the target hole as shown in Figure 1 (*p* = 0.0189, repeated measures ANOVA). AAV9-POMT2 treatment did not change the performance of wildtype animals (Figure 6A, *p* = 0.962, repeated measures ANOVA). By contrast, the latency to enter the target hole was reduced in AAV9-POMT2-treated knockout mice when compared to AAV9-EGFP-treated knockout mice (Figure 6B, *p* = 0.0226, repeated measures ANOVA). Similarly, AAV9-POMT2-treatment decreased primary latency to the target hole in the knockout mice (data not shown). As a control, the walking speed of these mice on the Barnes maze was also measured and not found to be statistically different (Figure 6C). There was no significant difference in time spent in the open arms or closed arms on the elevated plus maze between the different groups either (Figure 6D), indicating no significant changes in anxiety. During the probe trial on the Barnes maze (Figure 6E), the knockout mice poked the target hole and the two holes adjacent to the target hole (−1 and +1) fewer times than the wildtype mice did. However, AAV9-POMT2-treated knockout mice poked the target hole and the two holes adjacent to the target hole more times than AAV9-EGFP-treated knockout mice. These results indicate that spatial learning deficits in cerebral cortex-specific POMT2 knockout mice are ameliorated by postnatal AAV9-POMT2 treatment despite the finding that severe neuronal ectopia is not corrected.

**Figure 7:**
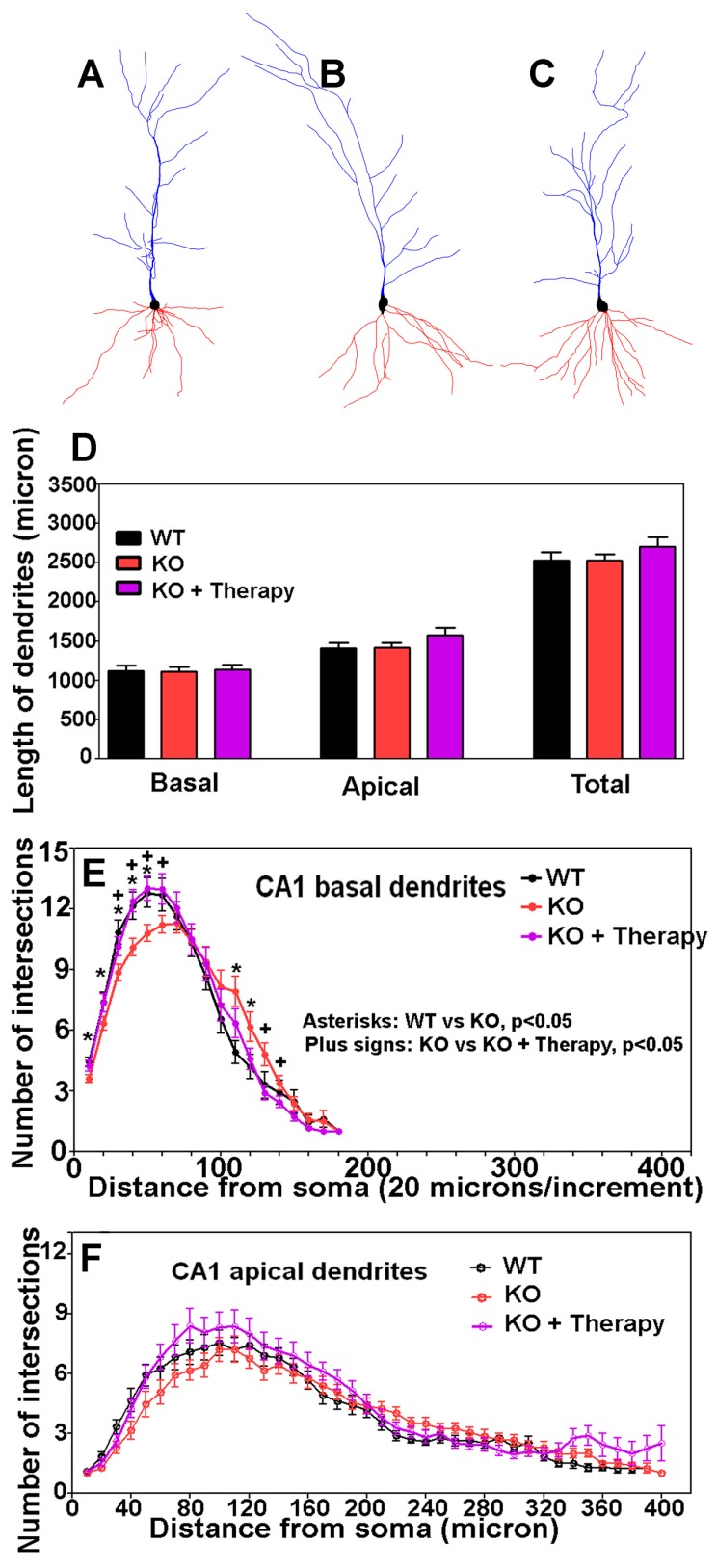
CA1 neurons of cerebral cortex-specific POMT2 knockout mice exhibit minor arborization changes in the basal dendrites rescued by postnatal gene therapy. Golgi staining was carried out on five- to six-month old mouse brains. Dendritic arborization of CA1 pyramidal neurons was evaluated by Sholl analysis. (**A**–**C**) Examples of Neurolucida drawing of CA1 pyramidal neurons from the wildtype, knockout, and AAV9-POMT2-treated knockout mice, respectively. The morphology of the CA1 neurons in the knockout mice appeared normal. (**D**) Lengths of basal and apical dendrites were equivalent between the three groups. (**E**) Sholl analysis of basal dendrites. The curve for the knockout mice (red color) shifted to the right when compared to the wildtype (black), indicating that basal dendrites in cerebral cortex-specific POMT2 knockout mice had fewer intersections near the soma (up to 60 μm) (compare red to black) but exhibited increased intersections further away from the soma (around 120 μm). Interestingly, the aberrant arborization of basal dendrites in the knockout mice was rescued by gene therapy (purple); (**F**) Sholl analysis of apical dendrites. Cerebral cortex-specific POMT2 knockout mice did not show significant differences in the number of intersections. Abbreviations: KO, knockout; WT, wildtype.

Learning deficits are usually associated with faulty dendritic spines. To determine whether dendritic spines had been affected in cerebral cortex-specific POMT2 knockout mice, we analyzed dendritic arborization and spine morphologies of CA1 pyramidal neurons by Golgi staining. Examples of camera lucida tracings of CA1 neurons from AAV9-EGFP-treated wildtype mice, cerebral cortex-specific POMT2 knockout mice, and AAV9-POMT2-treated knockout mice are shown in Figure 7A–C, respectively. There was no significant difference in the lengths of basal, apical and total dendrites, indicating that overall dendritic growth was not affected in the knockout mice (Figure 7D). However, Sholl analysis revealed that there was a rightward shift in the number of intersections for the basal dendrites in the knockout mice (Figure 7E), indicating that branching of basal dendrites was reduced proximal to the soma but increased distal to the soma in cerebral cortex-specific POMT2 knockout mice. These results suggested minor changes in wiring patterns of basal dendrites with incoming axon fibers. Interestingly, the basal dendritic branching defect was corrected in AAV9-POMT2-treated knockout mice. Sholl analysis did not reveal significant changes in the apical dendrites in the knockout mice (Figure 7F).

**Figure 8:**
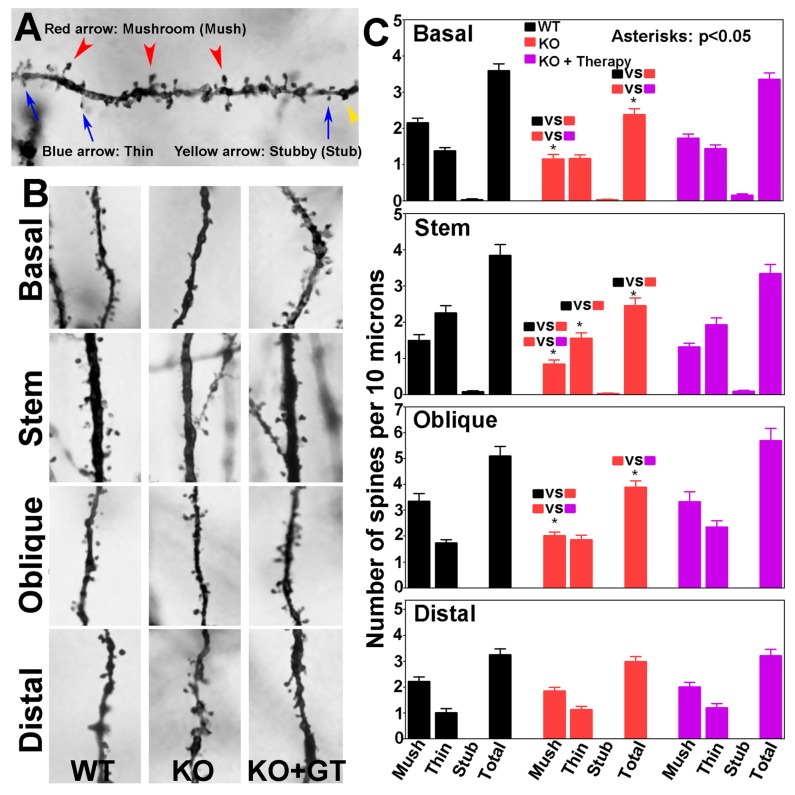
CA1 pyramidal neurons of cerebral cortex-specific POMT2 knockout mice exhibit reduced dendritic spine density which was rescued by postnatal gene therapy. The brains of five- to six-month old cerebral cortex-specific POMT2 knockout mice were processed for Golgi-staining. Spines on basal and apical dendrites, including those on the apical stems, of CA1 pyramidal neurons were counted. (**A**) Examples of mushroom, thin, and stubby spines; (**B**) representative segments of basal, stem, oblique, and distal dendrites in wildtype mice (**left**), knockout mice (**middle**), and AAV9-POMT2-treated knockout mice (**right**); and (**C**) number of mushroom, thin, stubby, and total spines per 10 μm lengths of basal dendrites, apical stem, and apical oblique and distal branches. Note mushroom spines were reduced in basal dendrites, apical stem, and oblique branches of the knockout mice. This defect was rescued in AAV9-POMT2-treated knockout mice. Thin spines on apical stems were also reduced in the knockout mice. Thus, the reduced spine density in the knockout mice was mainly caused by reduction of mushroom spines. Interestingly, AAV9-POMT2 treated knockout mice showed no significant difference from the wildtype mice indicating that postnatal gene therapy rescued faulty spines. Spine density on distal branches was not affected in the knockout mice, and was a feature that was not affected by gene therapy. These results indicate that O-mannosyl glycosylation deficiency affects dendritic spine morphology which is largely corrected by postnatal gene therapy. Abbreviations: WT, wildtype; KO, cerebral cortex-specific POMT2 knockout; KO+GT, AAV9-POMT2 treated knockout mice.

We determined the density of dendritic spines, as well as the density of spines with various morphologies, including mushroom, thin, and stubby types. Examples of mushroom, thin, and stubby spines are shown in Figure 8A. Representative segments of basal dendrites, apical stem, and oblique and distal branches of apical dendrites from the wildtype (left panels), knockout (middle panels), and AAV9-POMT2-treated knockout mice (right panels) are shown in Figure 8B. Total spine density on basal, stem, and oblique branches was lower in the knockout mice when compared to the wildtype mice (Figure 8C). The reduction of total spine density in the knockout mice was mainly due to the reduction of the mushroom type. Thin spines were significantly reduced on stems but not on other dendrites. Interestingly, the reduction of mushroom spines and total spines was rescued after postnatal gene therapy. There was no significant difference in the numbers of mushroom, thin, and stubby spines and the number of total spines between wildtype and AAV9-POMT2-treated knockout mice. Reduction in spine density in the knockout mice was reflected throughout the entire dendritic length, as revealed by the comparison of spine density on every 25-µm segment of basal and apical stem dendrites from the soma. Together, these results indicate that cerebral cortex-specific POMT2 knockout mice exhibit dendritic spine defects and that these defects are rescued by postnatal gene therapy.

## 4. Discussion

Several neuronal ectopic abnormalities including lamination defects in the neocortex, fused hemispheres, and a dentate gyrus with abnormal morphology were observed in cerebral cortex-specific POMT2 knockout mice [28]. In this report, we showed that these knockout mice also exhibited dendritic spine defects in CA1 neurons. The knockout animals showed increased latency to the target hole in the Barnes maze, indicating learning deficits. Interestingly, postnatal gene therapy to restore POMT2 expression rescued the dendritic spine defects and improved performance on the Barnes maze despite the fact that the neuronal ectopia phenotypes were not rescued.

It will now be of great interest to decipher the cellular and molecular mechanisms involved in spatial learning deficits and functional rescue after postnatal gene therapy. *O*-mannosyl glycosylation is found in a number of plasma membrane proteins including α-DG, receptor tyrosine phosphatase zeta/beta [50,51], the light chain of IgG2 [52], CD24 [53], and neurofascin [54]. Cadherins and plexins are recently found to be substituted by hexoses [55]. Cadherin-13 is confirmed to be *O*-mannosylated by α-mannosidase treatment [56]. Whether other cadherins and plexins are *O*-mannosylated will require confirmation from further carbohydrate analysis. O-mannosyl glycans on α-DG are required for α-DG to interact with laminin G domains of extracellular matrix molecules. Interestingly, dystroglycan is present at the synapses in the brain and long-term potentiation is diminished in the hippocampus of brain-specific DG knockout mice [57]. Furthermore, α-DG interacts with the synaptic proteins such as neurexins in the brain [45] and pikachurin in the retina [47,48,49]. Mutations of neurexin-1 result in deficits in associative learning in Drosophila [58] and long-term facilitation in Aplysia [59]. This study demonstrates that spatial learning deficits are correlated with faulty dendritic spines. It will be interesting to determine the *O*-mannosyl glycosylated proteins(s) and the extracellular matrix molecule(s) responsible for the dendritic spine defects in the absence of *O*-mannosyl glycosylation. We speculate that *O*-mannosyl glycosylation of multiple proteins are involved in regulation of dendritic spines.

It is of some interest to note that spine density in the distal branches of CA1 pyramidal neurons was not affected in the knockout mice. AAV9-POMT2 treatment did not alter the spine density in distal branches either. Distal branches of CA1 pyramidal neurons receive axonal inputs mainly from the perforant path fibers which originate in the entorhinal cortex. Basal dendrites receive inputs mainly from other pyramidal neurons, septal fibers, and commissural CA3 fibers from the contra-lateral hippocampus. Oblique branches receive axonal inputs mainly from the Shaffer collaterals of CA3 neurons. Reduction of spine density in basal and oblique dendrites but not distal branches suggests that *O*-mannosyl glycosylation deficiency affects synaptic inputs to CA1 pyramidal cells from CA3 neurons more so than perforant path axons.

Although it is presumed that neuronal ectopia contributes to the learning deficits, these data indicate that neuronal ectopia is not the sole cause of brain dysfunction and that spatial learning deficits found in the knockout mice can be corrected by restoring gene expression despite histological defects. We assume that dendritic spine defects may contribute to learning deficits and that recovery of dendritic spine defects may be involved in functional rescue after gene therapy. Interestingly, deletion of dystroglycan in postmitotic neurons does not cause neuronal ectopia but affects functional innervation of cholecystokinin-positive basket cell terminals on pyramidal neurons [60], supporting the pathological complexity of type II lissencephaly. Future studies will need to determine whether dendritic spine defects exist in other parts of the mutant brain including the neocortex, whether there are other neuronal wiring abnormalities in the mutant brain, whether electrophysiological functions of neurons are impacted not only in the hippocampus but also in the neocortex in mutant animals, and the effects of postnatal gene therapy on these parameters. Because of ethical and technical issues with prenatal gene therapy, postnatal gene therapy provides a promising avenue of treatment even though defective developmental brain histological structures are not corrected. This approach could be broadly useful in other types of neuronal migration disorders.

## Figures and Tables

**Table 1:** Abnormal distribution of wisteria floribunda lectin (WFA)-labeled neurons in the knockout neocortex is not rescued by postnatal gene therapy.

Genotype	WFA-Labeled Neurons in Compartments 1–5 (Mean ± SEM) (see Figure 5G–I)				
	1	2	3	4	5
Wildtype	0.8 ± 0.23	19 ± 3.57	13.2 ± 2.59	12.8 ± 2.59	3.2 ± 0.78
KO + PBS	4.2 ± 1.09 *	17.4 ± 3.31	11.4 ± 2.15	12.4 ± 2.39	4.6 ± 1.18
KO + AAV9-POMT2	4.4 ± 1.35 ** #	16.2 ± 3.11	11.8 ± 2.28	12.0 ± 2.42	4.8 ± 1.12

* *p* = 0.021 compared to wildtype; ** *p* = 0.045 compared to wildtype; # *p* = 0.83 compared to KO + PBS.

## Abbreviations

The following abbreviations are used in this manuscript:

AAV	adeno-associated viral vector
DG	dystroglycan
EGFP	enhanced green fluorescence protein
FKRP	fukutin-related protein
GT	gene therapy
LARGE	like-glycosyl transferase
PBS	phosphate-buffered saline
POMGnT1	protein O-mannose N-acetylglucosaminyl transferase 1
POMT2	protein O-mannosyl transferase 2
PVDF	polyvinylidene fluoride
TBST	Tris-buffered saline
WFA	wisteria floribunda lectin
WGA	wheat germ agglutinin
